# More negative self-esteem and inferior coping strategies among patients diagnosed with IBS compared with patients without IBS - a case–control study in primary care

**DOI:** 10.1186/s12875-015-0225-x

**Published:** 2015-01-28

**Authors:** Ewa Grodzinsky, Susanna Walter, Lisa Viktorsson, Ann-Kristin Carlsson, Michael P Jones, Åshild Faresjö

**Affiliations:** 1 Department of Medicine and Health, Unit of Research and Development, County Council of Östergötland, Linköping, Drug Research, Linkoping University, Linköping, Sweden; 2 Primary care, County Council of Östergötland, Linköping, Sweden; 3 Psychology Department, Macquarie University, Sydney, NSW Australia; 4 Institution of Clinical and Experimental Medicine, Faculty of Health Sciences, Division of Gastroenterology, Linköping University, Linköping, Sweden; 5 Department of Medicine and Health, Community Medicine, Faculty of Health Sciences Linköping University, SE-581 83 Linköping, Sweden

**Keywords:** Primary care, IBS, Self-esteem, Coping, Psychosocial factors

## Abstract

**Background:**

Irritable Bowel Syndrome (IBS) is a chronic, relapsing gastrointestinal disorder, that affects approximately 10% of the general population and the majority are diagnosed in primary care. IBS has been reported to be associated with altered psychological and cognitive functioning such as mood disturbances, somatization, catastrophizing or altered visceral interoception by negative emotions and stress. The aim was to investigate the psychosocial constructs of self-esteem and sense of coherence among IBS patients compared to non-IBS patients in primary care.

**Methods:**

A case–control study in primary care setting among IBS patients meeting the ROME III criteria (n = 140) compared to controls i.e. non-IBS patients (n = 213) without any present or previous gastrointestinal complaints. The data were collected through self-reported questionnaires of psychosocial factors.

**Results:**

IBS-patients reported significantly more negative self-esteem (p < 0.001), lower scores for positive self-esteem (p < 0.001), and lower sense of coherence (p < 0.001) than the controls. The IBS-cases were also less likely to report ‘good’ health status (p < 0.001) and less likely to report a positive belief in the future (p < 0.001). After controlling for relevant confounding factors in multiple regressions, the elevation in negative self-esteem among IBS patients remained statistically significant (p = 0.02), as did the lower scores for sense of coherence among IBS cases (p = 0.04).

**Conclusions:**

The more frequently reported negative self-esteem and inferior coping strategies among IBS patients found in this study suggest the possibility that psychological therapies might be helpful for these patients. However these data do not indicate the causal direction of the observed associations. More research is therefore warranted to determine whether these psychosocial constructs are more frequent in IBS patients.

## Background

Irritable Bowel Syndrome (IBS) is a chronic, relapsing gastrointestinal disease, characterized by abdominal pain and disturbed bowel habits. The diagnosis is based on symptoms and exclusion of organic gastrointestinal disease [1]. IBS affects approximately 10% of the general population and the majority is diagnosed in primary care [2]. It is associated with impaired quality of life and increased use of health care resources [3-5]. The etiology of IBS remains unclear and so far no specific biological abnormality has been identified that would explain the symptoms, despite intense research efforts [6]. Currently the pathophysiology of IBS is viewed as being caused by a dysregulation of the brain-gut axis [7,8] meaning disturbances in the bi-directional communication between peripheral factors in the gut such as neuro-immune mechanisms [9], mucosal barrier function [10], microbiota [11] on the one hand and the central nervous system [12-14] on the other. IBS has been reported to be associated with altered psychological and cognitive functioning [15] such as mood disturbances [16], somatization, catastrophizing [17] or altered visceral interoception by negative emotions and stress [18].

Stress has been reported to increase disease activity, with chronic stress likely to be more important than acute stress [19,20]. A recent study also showed that different aspects of social relationships and negative interpersonal interactions were associated with multiple aspects of IBS experience, such that negative social relationships marked by conflicts and negative exchanges were more consistently and strongly related to IBS outcomes than social support from the family and friends [21].

People use different coping strategies to manage illness and stress. These strategies can have positive or negative effects on their health status. Coping strategies using avoidant behavior are characterized by tendency to escape rather than managing difficulties. This behavior is related to increased self-blame and could lead to poor psychological adjustment i.e. lower coping ability [22]. Drossman and coworkers demonstrated in patients with IBS that illness behavior was the strongest predictor of the severity of functional bowel disorders [23]. In another study, patients with IBS were compared with non-IBS patients and healthy controls and in that study it was found that psychological factors were associated with the patient’s status rather than with the disorder per se [24]. Phillips et al. found four coping dimensions (active coping, instrumental support, self-blame, and positive reframing) differentiated IBS from healthy controls [25]. Patients with IBS have reported higher levels of self-blame than patients with inflammatory bowel disease (IBD) [22,26]. Coping can also be measured in terms of sense of coherence (SOC), a concept developed by Antonovsky [27]. SOC is defined as a global orientation that expresses the extent to which an individual has a pervasive, enduring, though dynamic feeling of confidence that life is comprehensible, manageable and meaningful [27]. This salutogenic theory focuses on why some people stay healthy despite stressful conditions. In addition, a strong SOC allows individuals to successfully cope with stressors.

A strong self-image is one of the key components for good health in general and affects the way people take care of themselves [28]. The self-image concept can be defined as the way a person behaves toward him or herself and the way in which a person chooses to act in relationships with other people. Self-image is a part of our social identity in which we become confirmed by other people [29]. A person’s self-image is shaped at an early age and continues to evolve and change throughout life. Self-image can also be measured in terms of self-esteem. In a study comparing IBS patients with IBD patients, lower self-esteem was found for the IBS group as well as a higher frequency of anxiety in relationships with others [20].

### Aims

We conducted this study with the aim of investigating the psychosocial constructs of self-esteem and sense of coherence among patients diagnosed with IBS compared with patients without any present or previous gastrointestinal complaints in a primary care setting. We hypothesized that individuals with IBS might have certain personality traits concerning lower self-esteem and inferior coping strategies than patients without IBS that make them more vulnerable for this disorder.

## Methods

### Study population and design

The study adopted a case–control design focusing on patients diagnosed with IBS i.e. IBS-cases in a defined region in south-east Sweden (The County Council of Östergötland). Ten Primary Health Care centres (PHCs), in the three major cities of the region, joined the study. These PHCs are responsible for primary care of a population of around 150 000 inhabitants (1/3 of the whole population in the total County Council). Of these three cities, one could be labeled as a white-collar city and two as blue-collar cities [30]. The selected ten PHCs were chosen based on defined criteria to ensure diversity concerning socioeconomic status, age of the population and number of immigrants.

**Figure 1:**
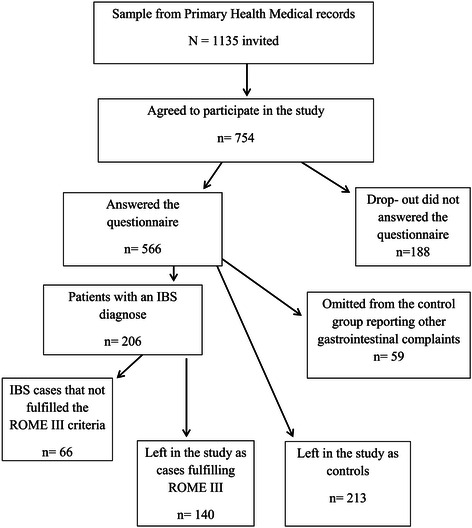
**Flow-chart of the study population.**

Subjects within the normal working age range (18–65 years) with a known IBS diagnosis and active symptoms during the last 2 years identified in the patient medical register of the selected PHC were invited to participate in the study. The control group comprised other patients at these health care centers with a similar age and sex distribution, although not matched 1:1, who sought care for other but less serious complaints not associated with gastrointestinal (GI) symptoms and with no GI diagnoses found in the patient register for the previous two years. Patients who agreed to participate after an invitation letter completed questionnaires and returned them by mail in pre-paid envelopes. A total of 1135 invitations were mailed out, of which 754 individuals agreed to participate, yielding an initial response rate of 66%. However 188 individuals who agreed to participate did not return a questionnaire, yielding a final response rate of 50%. A total of n = 140 IBS-cases who fulfilled the Rome III criteria agreed to participate in this study, and patients without IBS and any present or previous GI complaints comprise n = 213 controls. A flow chart of the participants in the study is shown in Figure 1.

### Questionnaires of psychosocial factors

Besides demographic measures and the ROME III questionnaire, questions about general health and belief in the future were asked, derived from The Swedish Living Conditions Survey of Health and Welfare survey. The questionnaire also included some psychosocial measurements: Sense of coherence and Structural Analysis of Social Behavior (SASB) [27,28], as well as selected questions: Influence on planning your work and important work tasks, from the demand-control questionnaire developed by Karasek [31]. Education was divided into three categories: low (primary school), medium (secondary or upper secondary school), or high (college or university). Marital status was dichotomized into the categories; living alone or married/de facto. Occupational status was divided into five categories; employed, unemployed, retired, on sick-leave or student. Questions about important tasks at work from the demand-control questionnaire were divided into three categories; important, neither or unimportant. Perceived health was dichotomized into two categories, good or poor health. Belief in the future was also dichotomized into two categories, optimistic view of the future or pessimistic about the future.

### Sense of coherence (SOC)

Sense of coherence is a theoretical construct explaining differences in how people perceive the world around them and thereby how they tend to cope with stressful and strenuous situations. This concept includes three main components; comprehensibility (the ability to understands what happens), manageability (to what extent the person were able to manage the situation) and meaningfulness (the ability to find meaning in the situation) [27]. This concept has been suggested as explaining how individuals cope with stressors in their lives. The higher the score, the more effective their coping strategy and the better their health outcome. The Swedish version of Antonovsky’s 13-item questionnaire (SOC-13) was used in this study [32]. Every item is scored on a Likert scale ranging from 1 to 7 points. Thus the total score ranges from 13–91 points. This 13-item version of SOC has been shown to be reliable, valid and cross-culturally applicable when evaluating how people can manage stress and still be healthy [33,34].

### Structural Analysis of Social Behavior (SASB)

The SASB model was developed and constructed upon interpersonal theory by Benjamin [35]. The central concept is “the self”. The self-image subscale of SASB consists of 36 items forming 8 domains representing how the individual relates to him or herself. The measurements comprise self-autonomy, self-affirm, self-love, self-protect, self-control, self-ignore, self-hate and self-blame. These are divided in two domains representing spontaneity versus self-control, three domains representing attachment and three domains representing disruption. These domains form a negative or a positive self-image related to overall feelings of self-worth or self-acceptance. High score indicates a high level of self-esteem, which involves self-respect and high self-worth. On the other hand, having low scores means low self-esteem and the individual feels inadequate, feels low self-worth and has low self-respect. The Swedish validated version of SASB used in this study [28] contains these 36 items/statements regarding agreement-disagreement on a VAS scale between “do not agree” (scale point 0) to “perfect agreement” (scale point 100). The calculation of the score was made using a syntax for SPSS.

### Ethical considerations

The study was approved by the Regional Ethical Research Committee at Linköping University (Dnr M41-09). Written informed consent was supplied by all participants.

### Statistical analysis

All data was stored in a common database and statistically analyzed using the SPSS version 21.0 software (SPSS Inc., Chicago, IL, USA). A comparison of IBS cases and controls with respect to all demographic and psychosocial variables is reported in Tables 1, 2 and 3. Descriptive statistics are reported as either counts and percentages within IBS case and controls for qualitative variables or as mean and standard deviation for quantitative variables (Tables 1, 2 and 3). Statistical inference was obtained via logistic regression for the odds of IBS and reported as odds ratios (ORs) along with 95% confidence intervals and p-values. OR values >1.0 indicate that the presence of or higher scores on that variable are generally associated with higher odds of IBS whereas OR values <1.0 indicate the presence of or higher scores on that variable are generally associated with lower odds of IBS. Differences between cases and controls for the quantitative psychosocial variables are reported as means and standard deviations and the p-values were calculated by unpaired *t*-test. In Table 4 we controlled for demographic variables which differed to a statistically significant extent between IBS and control groups to estimate the association of each psychosocial variable with IBS case/control status that was over-and-above such factors. Potentially relevant confounders in this analysis were age, civil status, educational level, health status and belief in the future. Separate multiple regressions were performed for each of the three psychosocial variables, since all these three were strongly correlated.

**Table 1:** **Sociodemographic data for IBS-cases and controls**

Sociodemographic data:	IBS (n = 140) n %	Controls (n = 213) n %	p-value
**Sex**			
Male	24 (17.1%)	40 (18.9%)	0.71
Female	116 (82.9%)	172 (81.1%)	
**Age**	46.7 (SD = 13.9)	51.4 (SD = 12.4)	0.001
**Social environment**			
White-collar city	69 (49.3)	118 (55.4)	0.32
Blue-collar cities	71 (50.7)	95 (44.6)	
**Marital status**			
Living alone	44 (31.7)	37 (17.5)	0.002
Married/cohabitant	95 (68.3)	174 (82.5)	
**Educational level**			
Low	19 (13.6)	15 (7.1)	0.049
Medium	69 (49.3)	95 (45.0)	
High	52 (37.1)	101 (47.9)

**Table 2:** **Characteristics of social data for IBS-cases and controls**

Sociodemographic data:	IBS (n = 140) n %	Controls (n = 213) n %	p-value OR (CI 95%)
**Occupational status**			(0.33)
Employed	91 (65.0)	156 (73.6)	1.00
Student	16 (11.4)	13 (6.1)	2.11 (0.97-4.59)
Retired	20 (14.3)	30 (14.2)	1.14 (0.61-2.13)
Sick leave	5 (3.6)	4 (1.9)	2.14 (0.56-8.18)
Unemployed	8 (5.7)	9 (4.2)	1.52 (0.57-4.09)
**Influence on planning the work**			(0.45)
Great influence	49 (49.0)	94 (57.3)	1.00
Some influence	42 (42.0)	60 (36.6)	1.83 (1.08-3.09)
No influence	9 (9.0)	10 (6.1)	1.73 (0.66-4.53)
**Important tasks at work**			(0.12)
Important	90 (91.8)	158 (96.9)	1.00
Neither	7 (7.1)	3 (1.8)	4.10 (1.03-16.23)
Unimportant	1 (1.0)	2 (1.2)	1.14 (0.10-12.74)
**Health status**			(<0.001)
Good	88 (62.9)	198 (93.8)	1.00
Poor	52 (37.1)	13 (6.2)	9.00 (4.66-17.37)
**Belief in the future**			(<0.001)
Believes in the future	93 (66.4)	184 (86.8)	1.00
Doubt about the future	47 (33.6)	28 (13.2)	3.32 (1.95-5.64)

**Table 3:** **Comparisons of means between IBS patients (cases) and non-IBS patients (controls) for the psychosocial indicators of negative- and positive self-esteem and sense of coherence respectively**

Psychosocial indicators:	IBS cases n = 140 mean (SD)	Controls n = 213 mean (SD)	p-value
Negative self-esteem	70.7 (52.8)	44.4 (39.0)	<0.001
Positive self-esteem	172.6 (47.9)	189.4 (43.7)	0.001
Sense of coherence	60.6 (11.7)	67.4 (9.6)	<0.001

**Table 4:** **Three separate multivariate analyses of the association between IBS cases and controls and negative, positive self-esteem and sense of coherence adjusted for other possible intervening factors**

	Negative self-esteem^a)^for cases and controls		Positive self-esteem^b)^for cases and controls		Sense of coherence^c)^for cases and controls	
	Standardized coefficient B	p-value	Standardized coefficient B	p-value	Standardized coefficient B	p-value
Age (linear trend)	-.12	0.01	.09	0.15	.23	<0.001
Marital status (Married v Single)	.03	0.61	.03	0.67	-.04	0.43
Education (linear trend)	-.09	0.17	.08	0.18	.11	0.03
Health Status (Poor v Good)	.05	0.43	-.08	0.21	-.14	0.01
Belief in the future (Doubts v Believes)	.37	<0.001	-.34	<0.001	-.43	<0.001
IBS case vs. controls	.13	0.02	-.05	0.39	-.10	0.04

^a)^Regression model adj R^2^ = .22, df = 6 F = 17.1 p < 0.001.

^b)^Regression model adj R^2^ = .15, df = 6 F = 11.1 p < 0.001.

^c)^Regression model adj R^2^ = .36, df = 6 F = 25.1 p < 0.001.

## Results

For the total sample of N = 353 IBS-cases and controls (see Table 1), there was a predominance of females for both cases and controls and the mean age was 46.7 (SD = 13.9 years for IBS cases and 51.4 (SD = 12.4) years for controls. Overall 76% of the participants in this study were married or cohabiting. Medium or high level of education were reported for around 86% of the cases and for 93% of the controls respectively. In this sample, 65% of cases and 74% of the controls were employed (see Table 2). Most subjects felt they had high or at least some influence on their work and also felt that their work was important.

IBS cases and controls did not differ significantly with respect to sex-ratio, social environment, occupational status, influence on planning their work or perception of the importance of their work. But, IBS cases tended to be younger than the controls and were less likely to be married or cohabiting and also tended to have a lower educational background. The IBS cases were less likely to report ‘good’ health status and were also less likely to report a positive belief in the future in comparison to the controls, see Table 2.

Table 3 indicates that the IBS cases on average scored higher on the negative self-esteem measure than controls and lower on the positive self-esteem measure. The IBS-cases’ average scores on sense of coherence were also lower than that reported by the controls, see Table 3. In addition, measurement of self-esteem was also significantly associated with educational level (positive r = 0.11, p = <0.001; negative r = −0.10, p = <0.001).

Three separate multiple regression analyses were undertaken for the association between IBS cases and controls for negative and positive self-esteem as well as sense of coherence, see Table 4. In all three analyses adjustments were made to control for relevant confounding factors. Table 4 indicates that IBS cases was significantly associated with elevated negative self-esteem and worse sense of coherence, but not with positive self-esteem after controlling for confounders. Several demographic factors, apart from IBS status, were also found to be associated with negative and positive self-esteem and sense of coherence (Table 4). Individuals who were pessimistic about the future tended to have higher levels of negative self-esteem but lower levels of positive self-esteem (Table 4). Older individuals tended to have lower levels of negative self-esteem but higher levels of sense of coherence (Table 4). Individuals with higher educational attainment and those with better health status tended to have higher sense of coherence (Table 4).

## Discussion

The results of this study support a hypothesis that individuals with IBS have certain personality traits concerning lower self-esteem and inferior coping strategies than patients without any present or previous GI complaints. In this study we found that IBS cases had higher levels of negative self-esteem but lower levels of positive self-esteem (Table 3). The higher levels of negative self-esteem remained statistically significant after controlling for a number of potentially confounding variables but the deficit in positive self-esteem did not remain (Table 4). Finally, IBS cases reported a lower coping ability, as expressed through the sense of coherence measure (Table 3), which remained statistically significant after controlling for potentially confounding variables (Table 4).

Self-esteem is a personality variable referring to the degree to which an individual values and accepts him or herself [35-37]. Earlier studies have shown that individuals with low self-esteem have more difficulties in responding to certain types of information regarding acceptance, rejection, evaluation or information that sounds threatening and blame themselves to a greater extent after a failure or mistake. The consequence of this behavior could be a focus on negative feedback rather than positive [38,39]. This particular behavior might be experienced as a stressor that triggers or worsens IBS symptoms. One can only speculate, if lower education can also mirror such negative self-esteem. In the present study we found that higher education was positively associated with positive self-esteem and that IBS cases tended to have lower educational attainment than controls, this might also be a reason for the observed excess of negative self-esteem among IBS cases. Some studies of health differences between occupational groups have found that these differences between IBS cases and controls were mainly explained by differences in sex ratio [40]. Mood disturbances such as depression and anxiety have previously been reported to be overrepresented among patients with IBS [23,41]. The present study supports the results of Bengtsson et al. demonstrating that IBS cases tend to have lower self-esteem compared with other patients, especially when it comes to close relationship with other people, they felt more insecure and anxious [20]. In another study both patients with IBS and IBD in comparison with controls relied on less contemplative problem solving and more on escape-avoidance strategies [42]. Individuals with a positive self-esteem are described as generally successful and probably divert their attention away from negative feedback about themselves, and are also more confident and optimistic [39]. As seen in the present study, the controls i.e. patients without GI-problems tended to have a more positive approach and a more optimistic belief in the future.

The total score of sense of coherence was slightly, but significantly, lower in IBS cases compared with controls, even when controlling for possible confounding variables. A limited number of studies have examined sense of coherence in patients with IBS compared to controls without IBS [43,44]. The present study is concordant with the consensus from these publications that patients with IBS have a lower sense of coherence than individuals without IBS.

Stress is known as a predictor for worsening of IBS symptoms and it may also contribute to inducing the disorder [45]. Living with a chronic disorder such as IBS, causes many everyday problems such as predicting and controlling symptoms that might be very problematic. A strong sense of coherence might focus on how to live with and cope with the disease instead of the impact of stressors that worsen the disorder [44]. Living everyday with IBS requires coping strategies to manage the daily symptoms. These strategies are used to manage conflict and illness and can have positive or negative (self-control, self-blame and escape) effects on health status. The symptoms of IBS may influence the individual’s coping strategy. It is also likely that factors such as worry, fear and feeling of isolation regarding the illness contribute to this diversity of coping strategies [41]. Crane et al. have suggested that the use of passive behavioural coping strategies among patients with IBS can be predicted as a putative consequence of illness-related social learning occurring during childhood, which may influence the development of habitual illness behaviour. The non-life threatening nature of IBS might make the sufferers more reliant on passive coping strategies to adjust to this discomfort [42,46].

One must of course raise the basic question of whether the presence of more negative self-esteem, and lower coping are personality traits that existed prior to the development of IBS symptoms or an outcome of the disorder? On the other hand, social learning behaviour, affiliation and social environment are grounded in early childhood [47]. It has been reported in previous studies that patients with IBS tend to have more negative early life events such as physical, sexual and emotional abuse than i.e. patients with organic GI-diseases [48-50]. IBS cases in the present study also reported more life events (divorce, serious illness etc.) than the controls (data not shown).

Good or poor health might also play a role and reflect self-image. As found in the present study, IBS cases reported poor health to a greater extent than controls. This might reflect more co-morbidity or be an outcome of the disease itself. In a previous study it was shown that patients with IBS were more burdened by co-morbidity and worry about serious diseases than healthy controls in an 8-year follow-up [51]. One can hypothesize that this image of poor health, more general disease complaints and even negative self-esteem might result in a less optimistic view about the future, as we also see in the present study. One has to take into consideration when interpreting these data that the majority of the IBS cases were 45 years and above, which might have an influence on all the variables measured. A younger study population might have reported these psychosocial factors differently, and more research among young adults is warranted in this field. However, the main results in the present study strengthen the well-established bio-psycho-social perspective about the etiology of this disorder [52,53]. To date there is no standardised treatment for this patient group so cognitive behaviour therapy (CBT) could be the most beneficial for IBS-patients in primary care. Several studies show that fear of symptoms and behavioral pattern of control and avoidance, is associated with decreased quality of life as well as worsening symptoms [54,55]. In psychological treatments, the most prominent intervention to reduce fear is exposure therapy, which is often part of CBT. Several studies of exposure-based CBT for IBS demonstrated that it is superior to attention control conditions [55,56] and that symptom improvements are mediated through change in symptom-related fear [57]. Studies of internet-delivered CBT have been performed for several somatic/functional and psychiatric disorders, with treatment effects similar to those obtained in studies of face-to-face CBT [58]. Research has shown that internet-delivered CBT based on exposure is an effective treatment for adults with IBS in terms of both increased quality of life and symptom relief [55,56], with sustained effects 18 months after treatment [59] as well as cost-effectiveness [60]. While internet-CBT can be offered to IBS patients in much larger scale than traditional face-to-face psychological treatments, the self-referred [55,56,61] and tertiary care [62] samples in these studies make it difficult to estimate the effect of the treatment for the typical IBS patient. However, before a large-scale implementation of internet-CBT, the effects of the intervention need to be investigated in a primary care population, where most IBS patients are treated.

Our study has strengths and limitations. One strength is that we used established and validated questionnaires. A possible limitation in using IBS diagnoses set in PHCs as we have done in this study, is the dependence on the general practitioner’s ability to make the correct diagnosis. However, studies have shown that general practitioners rarely misdiagnose IBS in particular [63,64]. There could, on the contrary, be a tendency to under diagnose these complaints in PHC. We used Rome III criteria (RIII) to verify the diagnoses and only the IBS cases that met the RIII were included in this study. Another possible limitation could be the use of self-reported data from questionnaires. A well-known phenomenon to take into consideration when using self-reported data is recall bias, but in general, self-reports are quite reliable and well established [65].

## Conclusions

The more frequently reported negative self-esteem and inferior coping strategies among IBS patients found in this study suggest the possibility that psychological therapies such as cognitive behavior therapy might be helpful for these patients. However these data do not indicate the causal direction of the observed associations. More research is therefore warranted to determine whether these psychosocial constructs are more frequent personality traits in IBS patients or if the disease itself lowers self-esteem and leads to inferior coping strategies.

## Abbreviations

CBT: Cognitive behavior therapy
IBS: Irritable bowel syndrome
IBD: Inflammatory bowel disease
SOC: Sense of coherence
SASB: Structural analysis of social behavior
PHC: Primary health care centers
GI: Gastrointestinal
VAS: Visual analog scale
